# Editorial: Radiomics Advances Precision Medicine

**DOI:** 10.3389/fonc.2022.853948

**Published:** 2022-03-02

**Authors:** Bo Gao, Di Dong, Huimao Zhang, Zaiyi Liu, Seyedmehdi Payabvash, Bihong T. Chen

**Affiliations:** ^1^ Department of Radiology, The Affiliated Hospital of Guizhou Medical University, Guizhou, China; ^2^ Institute of Automation, Chinese Academy of Sciences, Beijing, China; ^3^ Department of Radiology, First Hospital of Jilin University, Changchun, China; ^4^ Department of Radiology, Guangdong Provincial People’s Hospital, Guangdong Academy of Medical Sciences, Guangzhou, China; ^5^ Department of Radiology and Biomedical Imaging, Yale School of Medicine, New Haven, CT, United States; ^6^ Department of Radiology, City of Hope Comprehensive Cancer Center, Duarte, CA, United States

**Keywords:** radiomics, radiogenomics, precision medicine, cancer treatment, biomarker

Radiomics applies quantitative methods to medical images and derives phenotypic information that might not be obvious during traditional visual inspection. The potential for radiomics to identify previously unrecognizable imaging biomarkers for tumor genotype and pathology has sparked considerable interest in exploring the clinical potential of radiomics (1). Since its initial presentation in 2012 (2), radiomics has been widely applied in oncology (Wu et al.) and has achieved robust performance in assessment of genomic features (Shen et al.) (3); tumor subtypes (4, 5); nodal metastases of various cancers such as colorectal cancer, gastric cancer, breast cancer (6–8) and occult distant metastases (9). In addition, radiomics has been used in evaluation of treatment response (10, 11) and outcome prediction (12, 13). The recent rapid development of artificial intelligence and its application in mining medical “big data” have further advanced the field of radiomics in both oncological research and clinical practice.

Precision medicine refers to tailoring treatments to patient-specific information, such as genomic makeup and molecular characteristics, to optimize the treatment strategy for each patient (14). Radiomics can identify tumor features that reflect the underlying tissue characteristics for each patient and can inform precision medicine (15). The field of radiomics has already accomplished a great deal toward promoting personalized medicine and assisting clinical decision-making. However, there are still several issues preventing its wide application for precision medicine. These issues include challenges with the interpretability, reproducibility and biological correlation of radiomic features. In addition, data mining and processing techniques for radiomic analysis need improvement.

Therefore, we launched this Research Topic, “*Radiomics-Based Tumor Phenotyping in Precision Medicine*”, to provide a platform for reporting radiomic studies focusing on precision medicine. We received more than 60 manuscripts focused on various tumors throughout the body, including brain glioma, brain metastasis, nasopharyngeal carcinoma, lung cancer, breast cancer, gastric cancer, renal cancer, rectal cancer, and prostate cancer. After peer review, 43 papers were selected for publication with this Research Topic in Frontiers in Oncology. Among the cancers studied, lung cancer stood out as having the most publications, with 11 papers. In addition, more than half of the papers were focused on diagnosis (Zhang et al.), staging (Zhou et al.), and genetic prediction (Song et al.). Response to immunotherapy, which is a hot topic in oncology research, was studied in two papers (Shen et al.) (Liu et al.). Furthermore, a wide range of computational methods, such as conventional radiomics, deep learning (Zhang et al.), delta-radiomics (Ma et al.), and intra-peritumoral radiomics (Li et al.), were reported in this Research Topic. For instance, Zhang et al. developed a multi-parametric MRI radiomic model to differentiate clinically significant and insignificant prostate cancer. The authors evaluated 159 patients with prostate cancer from two centers for radiomic features extracted from MRI, including a T2-weighted sequence, diffusion-weighted imaging results, and apparent diffusion coefficient (ADC) images. Minimum-redundancy maximum-relevance (mRMR) and least absolute shrinkage and selection operator (LASSO) analysis methods were used to select key MRI features. Their work showed that the model combining the radiomic signature and ADC values produced better classification performance than either model alone.

Regarding radiogenomic analysis, Song et al. conducted a CT radiomic study for predicting anaplastic lymphoma kinase (ALK) mutation in patients with lung adenocarcinoma. This study retrospectively analyzed 335 patients with lung cancer from a single center and developed three models (radiomic, radiological, and integrated models). Their integrated model, which combined radiomic features, conventional CT features, and clinical features, achieved the best performance for predicting ALK mutation in patients with lung cancer. In another study of lung cancer, Chen et al. used lung CT radiomics to differentiate small cell lung cancer (SCLC) from non-small cell lung cancer (NSCLC) in 69 patients. The researchers built predictive models with a multilayer artificial neural network and their SCLC/NSCLC classifier achieved robust performance with an area under the curve (AUC) of 0.93.

A timely study of imaging biomarkers for predicting response to immunotherapy in advanced NSCLC was performed by Liu et al. The researchers retrospectively enrolled 197 patients with NSCLC from nine centers. Each patient had undergone immunotherapy with immune checkpoint inhibitors, such as anti-PD-1 therapies, and received follow-up assessment for treatment response (responder/non-responder). They found that a combined prediction model incorporating a delta-radiomic signature and a clinical factor (distant metastasis) performed well in distinguishing responders from non-responders with AUCs of 0.83 and 0.81 in the training and validation cohorts, respectively. This study indicated that delta-radiomics could be useful for identifying imaging biomarkers to assess the early response to immunotherapy in patients with NSCLC and facilitate precision medicine.

Jiang et al. developed a radiomic model to predict the stage, size, grade, and necrosis (SSIGN) score preoperatively in patients with clear cell renal cell carcinoma (ccRCC). The investigators enrolled 330 patients with ccRCC from three centers and placed them randomly into a training cohort and two external validation cohorts. A radiomic signature was built with the 16 selected image features from CT images acquired in the nephrographic phase. They found that the signature performed better than the image feature model constructed by intra-tumoral vessels (all *p* < 0.05) and showed similar performance to the fusion model integrating radiomic signature and intra-tumoral vessels (all *p* > 0.05) in terms of the discrimination in all cohorts. The radiomic signature showed promising results in predicting tumor aggressiveness in patients with ccRCC.

Feng et al. explored the correlation between PET/MRI radiomic features and the metabolic parameters in patients with nasopharyngeal carcinoma (NPC). All 100 NPC patients in the study underwent whole-body PET/MR examinations. Radiomic features from both the MRI and PET images, along with metabolic parameters from the PET images, were analyzed. To discriminate early-stage from advanced-stage NPC, they built MRI and PET models, which achieved reasonable performance with AUCs ranging from 0.69 to 0.90. They also showed correlations between the metabolic parameters and radiomic features of primary NPC based on PET/MRI.

This Research Topic, which contains a unique collection of radiomic studies, contributes important new information to the body of knowledge related to using artificial intelligence for precision medicine. We are encouraged by the great support from the research community; a total of 376 authors contributed to the 43 selected papers. In addition, this Research Topic has generated significant attention in the field, with over 87,000 views so far. Nevertheless, more work is needed to advance the field of computational imaging. First, most of the studies in this Research Topic were from a single center, which makes them prone to selection bias. Future large-scale multi-center studies should be performed to address the generalizability and to validate the results. Second, all studies in this Research Topic were retrospective, and may be limited by inherent confounding variables such as a heterogeneous study cohort, multiple different imaging protocols and scanners, and various imaging reconstruction methods. Lastly, we encourage sharing of radiomic data and artificial intelligence methods, which will undoubtedly facilitate the development of robust predictive modeling and imaging biomarkers to guide diagnosis and treatment in precision medicine.

## Author Contributions

BG wrote the original draft, assembled and incorporated comments from the co-authors, and crafted the final draft. All co-authors contributed to manuscript review and approved the final version.

## Funding

This work is supported by the National Natural Science Foundation of China (No. 81471645, 81871333), Guizhou Province 7th Thousand Innovational and Enterprising Talents (GZQ202007086), 2020 Innovation group project of Guizhou Province Educational Commission (KY [2021]017), Guizhou Province Science and Technology Project (No. Qiankehe Support [2020]4Y193, [2021]430), Guiyang Science and Technology Project (No. 2020-10-3).

## Conflict of Interest

The authors declare that the research was conducted in the absence of any commercial or financial relationships that could be construed as a potential conflict of interest.

## Publisher’s Note

All claims expressed in this article are solely those of the authors and do not necessarily represent those of their affiliated organizations, or those of the publisher, the editors and the reviewers. Any product that may be evaluated in this article, or claim that may be made by its manufacturer, is not guaranteed or endorsed by the publisher.
